# Natural Compounds and Their Potential in Eating-Related Aspects of Mental Health Disorders

**DOI:** 10.3390/nu17142383

**Published:** 2025-07-21

**Authors:** Wenbin Ma, Ralf Regenthal, Ute Krügel

**Affiliations:** 1 School of Health and Rehabilitation, Chengdu University of Traditional Chinese Medicine, Chengdu 611137, China; mawenbin@cdutcm.edu.cn; 2 Division of Clinical Pharmacology, Rudolf Boehm Institute for Pharmacology and Toxicology, Leipzig University, 04107 Leipzig, Germany; ralf.regenthal@medizin.uni-leipzig.de; 3 Rudolf Boehm Institute for Pharmacology and Toxicology, Leipzig University, 04107 Leipzig, Germany

**Keywords:** mental health disorders, eating disorders, *Rosmarinus officinalis*, *Ginkgo biloba*, *Bupleurum chinense*, berberine, gut-brain axis

## Abstract

**Background and Objectives**: Mental health and healthy eating are inextricably linked by bi-directional interaction. As pharmacological interventions for eating disorders and mental illness have limited efficacy and are associated with significant side effects, natural compounds traditionally used in these fields represent an extremely rich source for potential future drugs. This review aims to summarise complex and/or specific pharmacological and clinical effects of mixed compositions and individual compounds derived from *Rosmarinus officinalis*, *Ginkgo biloba*, and *Bupleurum chinense* as well as from *Berberis vulgaris* and other berberine (BBR)-containing plants, which have been traditionally used for eating and mental health purposes. **Results and Conclusions:** The data on favoured natural compounds and main ingredients of compound mixtures presented here could provide new impetus for preventive or targeted supplementary treatment, potential drug development, and the design of new compound congeners with improved target spectrum and potency in mental health disorders and eating-related issues. Contemporary methodological development steps in this direction are then proposed.

## 1. Introduction

Living in an ever-changing world requires ongoing human–environmental and interpersonal interaction for subjective and physical well-being, including processes such as recognition, evaluation, and reorientation. Mental health and healthy eating are inextricably linked. Psychological well-being strongly influences the quality and quantity of food consumed, but nutrition can also influence mental health [1]. This bi-directional interaction is widely experienced and accepted. Therapeutic strategies in this field, especially pharmacological intervention are very limited and response rates in both, eating disorders (ED) and mental illness are still unsatisfactory.

Genetic risk factors and epigenetic modulation by multiple environmental and social impacts, which are not fully understood, may contribute to the etiopathogenesis and pathophysiology of mental health problems and altered eating behaviour. In addition, body dissatisfaction and dieting associated with personality disorders often precede ED, i.e., anorexia nervosa (AN), bulimia nervosa (BN), binge eating disorder (BED), avoidant restrictive food intake disorder (ARFID), pica, and rumination disorder (RD) [2]. Sociocultural distress provoked by idealised body images, mobbing, and humiliation may promote the development of anxiety, mood disorders and depressive episodes and thus act as a booster for ED. Such distress can also alter the reward system, leading to overeating resulting in obesity (OB) or substance abuse. Notably, OB and ED are phenomenologically opposite. However, in their development, both seem to share features ranging from ambivalent to disturbed perception and cognition. Today, the events in the individual development of either mental health problems or ED are poorly understood and even less so the relationship between both.

Not surprisingly, psychotherapeutic interventions are the mainstay of treatment for ED, as there are very limited psychopharmacological options for these disorders, e.g., fluoxetine and topiramate for BN, olanzapine for AN, lisdexamfetamine and topiramate for BED. However, like most of psychopharmacological active drugs, these medications have significant side effects, including those on eating behaviour itself. Moreover, olanzapine and topiramate have not yet received marketing authorization for use in ED.

Due to the limitations of drug treatment in ED, it is justified to search for more effective and safer treatment strategies also exploring natural products that are traditionally used for mental well-being without known safety concerns.

The aim of this narrative review is to provide a summary of complex and/or specific pharmacological effects of selected compositions or individual compounds derived from medicinal plants, here *Rosmarinus officinalis*, *Ginkgo biloba*, *Bupleurum chinense*, and *Berberis vulgaris*. The idea behind this is to provide new impetus for targeted supplementation and for potential drug development by different enhanced and standardised compositions and for the design of new compound congeners with improved target spectrum and binding affinity.

## 2. *Rosmarinus officinalis*

*Rosmarinus officinalis*, a member of the family Lamiaceae, is originally native to the Mediterranean region. Like several other kitchen spices, rosemary is used in traditional ethnic communities for a wide variety of health purposes as reviewed by Borges et al. [3]. These include gastrointestinal, digestive and appetite disturbances, circulatory and blood pressure problems, inflammation-related diseases (rheumatism, asthma bronchiale), and improvements in mental health like depressed mood, anxiety, or memory.

In addition to its use as an intensely aromatic spice, rosemary slows down the rancidification (oxidation) of oils rich in polyunsaturated fatty acids [4,5]. As an antimicrobial preservative, it inhibits the growth of bacteria that contribute to food spoilage [6]. Recognising this, rosemary extract is approved as antioxidant INS (E) No. 392. The aerial parts of the plant are used either as tea, tincture, or as essential oil (ISO 1342:2012) [7]. The latter is composed of more than 150 compounds and is particularly rich in volatile anti-inflammatory monoterpenes such as camphor [8], α-pinene [9], and 1,8-cineole (eucalyptol) [3,10]. More than 35 phenolic compounds were identified in rosemary extracts: phenolic diterpenes (e.g., carnosic acid, carnosol, rosmanol), flavonoids (e.g., gallic acid, catechin, hesperitin, quercetin, kaempferol, apigenin), and phenolic acids such as caffeoyl derivatives (e.g., caffeic acid and its ester, rosmarinic acid) [11,12], all of which have antioxidant activity in in vitro bioassays [13,14]. Pentacyclic triterpenes (sapogenins) of lupine, oleanane, and ursane structures are also found. Many of these identified compounds are also components of other herbs used for health purposes.

The potential pharmacological principles of individual compounds or required specific compositions of rosemary preparations relevant to body weight and metabolic regulation, circulation stimulation, and neuroprotection in mental health are only partially understood to date [15].

However, some common applications of rosemary are directly related to its anti-inflammatory biological activity, like asthma and rheumatic diseases. There is evidence that chronic low-grade inflammation by unbalanced production of pro- and anti-inflammatory (adipo)cytokines contributes to obesity-related insulin-resistance [16]. Likewise, in humans, mental disorders, such as anxiety, depression, or ED, and various types of dementia, bipolar disorder, and schizophrenia are partly linked to inflammatory processes, which makes rosemary preparations attractive to exploit [17,18,19].

Apparently mediated by carnosic acid, a potent inhibitor of prominent digestive enzymes like lipase, α-amylase, and α-glucosidase, body weight and insulin levels were reduced in lean and obese Zucker rats [20,21]. Furthermore, an improvement in metabolic disease-associated weight gain, inflammatory markers in the liver, pancreas, and brain was reported in a high-fat diet (HFD)-induced mouse model following oral administration of carnosic acid, as evidenced by a decrease in pro-inflammatory cytokines [22]. In particular, the inflammatory response to HFD in mouse brain was associated with an activation of the nuclear factor kappa B (NF-κB) signalling pathway, which was attenuated by carnosic acid treatment. Similar observations were made for limonene, which counteracted elevated apoptosis, inflammation, and oxidative stress in mouse brain after HFD [23].

However, in a double-blind, randomised, placebo-controlled clinical trial (RCT) in which patients with non-alcoholic fatty liver disease were treated with rosemary leaf powder (4 g per day) or placebo as part of a weight-loss diet for eight weeks, no additional beneficial effect on primary and secondary outcomes were detected [24]. In another cross-over RCT in overweight men, the postprandial inflammation caused by hyperglycaemia and hyperlipidaemia after oral ingestion of a high-saturated-fat, high-carbohydrate meal was attenuated by undefined amounts of rosemary in a mixture with various other spices [25].

Chronically restrained mice that developed hypercorticosteronemia and impaired insulin signalling, accompanied by cognitive impairment, were treated with another rosemary ingredient, the lipophilic pentacyclic triterpenoid ursolic acid [26]. This compound not only attenuated stress-induced reduction in body weight but also reduced corticosterone and tumour necrosis factor α (TNF-α) concentrations in plasma and parameters of oxidative stress in brain. In addition to an improved insulin sensitivity, the cognitive decline in the stressed mice was attenuated too. Although the underlying molecular mechanisms for ursolic acid-mediated anti-inflammatory effects are not enlightened so far, the suppression of the transcription factor NF-κB-regulated gene expression modulating cytokine production and cell survival in response to stress is documented in vitro and in vivo in a mouse model of graft-versus-host-disease [27]. Similarly, ursolic and rosmarinic acid were reported to prevent amyloid Aβ_1–42_-induced deficits in cognition, synaptic regulation and adult hippocampal neurogenesis in mice [28]. Several other animal studies, although highly heterogeneous, confirmed beneficial effects of various preparations of rosemary in cognition and disease models [29].

The anti-inflammatory activity of mono- and diterpenes in rosemary is frequently reported [30]. Different but interacting pharmacological principles are proposed. One pathway involved is the inhibition of enzymes of the arachidonic acid cascade such as 5-lipoxigenase (5-LOX) and cyclooxygenases (COXs), which reduces the synthesis of inflammatory leukotrienes (LTB4) and prostaglandins (PGE2), e.g., by 1,8-cineole [31,32]. Another signalling pathway for some terpenes is the reduction in NF-κB activity. This attenuates the synthesis of the pro-inflammatory mediators TNF-α, interleukin-1β (IL-1β), and IL-6, of inflammatory enzymes (e.g., COX2, inducible nitric oxide synthase (iNOS)) and the expression of adhesion molecules and chemokines [33,34,35]. As a third mechanism, rosemary components, e.g., carnosol exert high antioxidant activity, thereby attenuating the harmful effects of reactive oxygen species (ROS) and lipid peroxidation [14].

These growing insights into the anti-inflammatory potential and the safe traditional use promote interest in rosemary for supportive application in mental disorders and ED. Preclinical observations in a mouse model of depression revealed an alleviated depression-like behaviour and a reduction in hippocampal oxidative stress by oral treatment with 1,8-cineole [36]. Preclinical mechanistic investigations on the central bioactivity of rosemary focused on neurotransmission. From behavioural studies with healthy mice fed acutely and sub-chronically with rosemary extract, Machado et al. (2009) predicted that rosemary’s antidepressant-like effects were based on interaction with the monoaminergic system [37]. In particular, these acute effects were evoked in a dose-dependent manner by the rosemary constituent ursolic acid, which involved dopamine D1 and D2 receptors [38]. In agreement with responses of human patients to antidepressants, no psychostimulant effects were detected after acute oral administration of different rosemary preparations and for isolated carnosol in a similar behavioural approach in mice [39].

The described preclinical data are consistent with the observed antidepressant-like effects of single doses of rosmarinic and caffeic acid, which did not affect either monoamine uptake into synaptosomes or mitochondrial monoamine oxidase activity in mouse brain [40]. However, Lin et al. (2015) found evidence for a rosmarinic acid-mediated reduction in serotonin turnover in the rat hippocampus [41]. Moreover, both acids acutely induced a dose-dependent reduction in defensive freezing behaviour in mice exposed to conditioned fear stress [42]. Similarly, in healthy mice, isolated rosmanol, cirsimaritin, and salvigenin elicited antidepressant- and anxiolytic-like behaviour, attributed to modulation of GABA-A receptors [30]. In a recent study, systemically applied carnosic acid, a selective KCNQ3/5 potassium channel opener in parvalbumin-positive GABA neurons, reduced cell excitability and thereby diminished the cocaine reward in mice [43]. Finally, anxiolytic-like effects of rosemary in mice appear to involve the stimulation of oxytocinergic transmission [44]. This is remarkable as oxytocin is strongly involved in the control of stress responses and food intake and contributes to adaptive active coping in animals and humans [45].

Whereas behavioural effects of rosemary or of some integral components were reproducible in essence in preclinical research, data on rosemary from clinical studies on mental health are scare. In an RCT with healthy Iranian college students, rosemary (500 mg twice daily for one month) improved prospective and retrospective memory and sleep quality, and in addition reduced anxiety and depression [46]. In Japanese adult working men diagnosed with a poor mental health score, a four-week intervention with oral rosemary ameliorated mental energy and sleep quality [47]. Plasma analysis before and after consumption of rosemary tea (5 g dry weight once daily) by healthy volunteers for 10 days revealed significantly higher concentrations of a reliable biomarker of depression, the brain-derived neurotrophic factor (BDNF), an effect that may be related to its signalling through the neurotrophic tyrosine kinase receptor 2 (TRKB), which is essential for potential antidepressant actions [48,49].

In a small group of patients with newly diagnosed major depression, dried plant powder, which was administered as add-on to selective serotonin reuptake inhibitor (SSRI) therapy, reduced anxiety and depression scores significantly more effectively than SSRI alone [50]. Interestingly, some patients of the rosemary group reported increased appetite as side effect. Today, although appetising properties for rosemary are often mentioned, there is no sufficient evidence for that.

To the best of our knowledge, no clinical data about rosemary administration in eating disorders (ED) are available so far. However, ED is strongly linked to poor gut microbiome health, dysbiosis, and inflammation, as well as to impaired microbiome metabolite signalling involving short-chain fatty acids and tryptophan via vagus nerve innervation to the brain [51,52,53,54]. So, there is evidence that an impaired bi-directional communication along the microbiota–gut–brain axis and a malfunction of central pathways regulating food intake, motivation, and execution, as well as hedonic aspects of food intake or its restriction, contribute to the pathology of ED. Similarly, the conception of mental illness has been strongly associated with alterations in intestinal microbiome [55,56,57,58,59]. Therefore, the development of microbiome-based (supporting) therapies in ED could be promising. Such strategies are already in therapeutic use, possibly unintentionally, as multiple antidepressants have antimicrobial effects too, and as on the other hand some antibiotics may mediate antidepressant responses [60,61,62].

Rosemary and its components provide an example of natural products with multiple biological activities that also comprise antimicrobial properties beyond its effect as a preservative for food. In a preclinical model of chronically restrained mice with a depression-like outcome, Guo et al. (2018) found a significant intestinal dysbiosis rated as reduced Shannon index, enhanced community abundance of *Bacteroidetes* and *Proteobacteria*, and lowered *Firmicutes* and *Lactobacillus* spp., along with inflammatory responses in serum and the hippocampus [63]. The concomitant oral administration of a rosemary extract containing 60% carnosic acid in this study not only attenuated depression-like behaviour and inflammatory surrogate markers but also re-balanced the gut microbiota. To date, the link between the rosemary-mediated modification of gut microbiota and its supposed antidepressant potential seen in acute predictive models is only poorly understood.

Apart from direct effects on gut microbiome, the overall biological activity of rosemary relies on the bioavailability of its constituents. Romo Vaquero et al. (2013) [64] evidenced intestinal uptake and subsequent appearance of metabolites of carnosic acid, carnosol, and rosmanol in plasma in substantial concentrations for several hours in Zucker rats. Furthermore, small quantities of carnosic acid and carnosol (along with some of their metabolites) were detected in rat brain tissue [64]. Plasma levels of both were in a magnitude that correspond to their biological activity in a low micromolar range.

Analysis of different in vivo toxicity studies reveals that even high doses of rosemary extracts, carnosic, or ursolic acid fed over 90 days were safe [65]. In 2016, the Joint FAO/World Health Organisation (WHO) Expert Committee on Food Additives (JECFA) stated no safety concerns and established a temporary acceptable daily intake (ADI) for rosemary extract according to its specifications of 0–0.3 mg/kg body weight, expressed as carnosic acid plus carnosol content. This considered a no-observed-adverse-effect-level (NOAEL) of 64 mg/kg per day [66]. Importantly, in later studies on reproductive and developmental toxicity, according to the test guidelines of the Organisation for Economic Cooperation and Development’s (OECD) and the specifications for food additives of the EU and JECFA, the NOAEL was doubled [67,68,69].

## 3. *Ginkgo biloba*

*Ginkgo biloba* L. is an endemic tree in China, grown worldwide today for nutrition, in particular the value of its seeds; for decoration; and also for health purposes. Its leaves, Ginkgo folium, are valued by the Anatomical Therapeutic Chemical/Defined Daily Dose Classification (ATC) of the WHO and the European Medicines Agency (EMA) as an antidementia drug (ATC N06DX02, Ginkgo folium) or as having “well-established use” for the improvement of (age-associated) cognitive impairment and of quality of life in mild dementia, respectively [70,71].

Evidence for the pharmacotherapeutic effects of natural products is frequently limited by their different phytochemical composition or product-by-process variations determining their pharmacokinetic and pharmacodynamic properties and therefore their clinical efficacy and safety.

A way to overcome these limitations was provided in case of *Ginkgo biloba* L. by strong defined growing and harvesting conditions and manufacturing processes providing standardised extracts, like EGb 761^®^ and LI 1370, with enriched content of various ginkgo-specific flavonol glycosides and terpene trilactones (ginkgolides, bilobalide) considered to be the primary pharmacologically active components [72].

Today, the data situation regarding beneficial effects in Alzheimer’s disease (AD) in clinical studies is still inconsistent. Whereas multiple studies reported beneficial effects of EGb 761^®^ treatment in AD and vascular dementia [73,74], in other RCTs, this extract does not seem to reduce the risk for the progression of AD or its incidence, nor did it result in less cognitive decline in older adults with normal or mild cognitive decline [75,76,77]. A more recent analyses of efficacy and methodological challenges of the defined extract mixture EGb 761^®^ in trials for mild-to moderate dementia did not consistently demonstrate its clinical utility for this indication [78,79]. Similarly, the National Center for Complementary and Integrative Health (NCCIH) of the National Institutes of Health (NIH) stated that there is no conclusive evidence that this defined *Ginkgo biloba* L. extract is helpful to improve or prevent cognitive decline [80]. Nonetheless, recognising overall data and focusing on beneficial effects on behavioural symptoms [81], EGb 761^®^ was adopted in the dementia guidelines in Germany [82].

It should be considered that about 110 flavonoids, several terpenoids and nor-terpenoids, alkylphenols and alkylphenolic acid, carbonylic acids, lignans, polyanthocyanides, polyprenols, and polysaccharides, ingredients of ginkgo, might act in concert by broad pharmacological spectra [83]. Dependent on individual baseline sensitivity conditions, i.e., severity with respect to cognitive performance and social functioning, the observed effects in dementia should be valuated with care [84].

Apart from being used to treat or prevent cognitive decline, ginkgo extracts have been reported to have beneficial effects in psychiatric disorders, in particular on anxious mood or generalised anxiety disorders, as well as attention-deficit hyperactivity disorder (ADHD), and as an adjuvant to antipsychotics in schizophrenia, thought to be related in particular to the antioxidant properties of ginkgo components [85,86,87]. A recent meta-analysis of 21 trials preferentially from China compared the effects and safety of ginkgo extracts on patients with depression [88]. Here, the scores like the Hamilton depression scale (HAM) and the modified Barthel index (MBI) were significantly improved in ginkgo-treated patients. Furthermore, significantly elevated serum levels of BDNF and serotonin (5-HT) in these patients suggested that this natural product could lower depression symptoms. However, these observed beneficial characteristics of ginkgo extracts must be reproduced in clinical trials with a larger numbers of patients, also outside of China to avoid ethnic and genetic biases, with a highly standardised study design, particularly with regard to indication and disease status, to compound composition, to dose, and to treatment regime.

In addition to antioxidant activity decreasing ROS, ginkgo components are proposed to exert anti-inflammatory effects [89], to modulate enzymes responsible for degradation of neurotransmitters and formation of amyloid plaques [90,91], and to modulate BDNF expression in the hippocampus of stressed rats, a key player in the neuroplasticity hypothesis of depression [92].

So far, as the authors have researched, clinical studies on the effects of ginkgo extracts on eating behaviour in eating or mental disorders are not available, though this aspect attracts increasing interest.

Preclinical studies provide some preliminary hints that ginkgo extracts or selected components of them may modulate eating-related emotions, appetite, and food choice, as well as body weight regulation. As an example, in rats, in which the bacterial endotoxin lipopolysaccharide (LPS) induced symptoms of anhedonia, a 14-day pretreatment with EGb 761^®^ prevented the decline of sucrose and food consumption and slightly alleviated the LPS-induced attenuation of dopamine in the reward-related nucleus accumbens [93,94]. Although the development of the animal’s body weight under EGb 761^®^ pretreatment alone is not reported, a comparatively higher food consumption of the EGb 761^®^ group was in line with lower body weight loss 24 h after LPS-induced sickness behaviour.

In healthy mice, a seven-day treatment regime with ginkgo extract, and specifically with the terpenoid ginkgolide A, produced anxiolytic-like behaviour without alterations in body weight [95]. Other ingredients, the ginkgolides B, C, or bilobalide did not alter body weight or the behaviour in a predictive anxiety test. Likewise, the body weight of healthy rats was not affected after a 15 day-treatment with EGb 761^®^ [96]. However, when rats with diet-induced obesity were treated with a ginkgo extract, the food intake and body adiposity were significantly reduced, whereas insulin sensitivity was increased, accompanied by a restored insulin-signalling cascade in muscle [97]. Compatibly, obese mice treated with a ginkgo extract expressed higher amounts of proteins associated with adipogenesis, carbon metabolism, and mitochondrial function and concomitantly showed a reduction in adipocyte hypertrophy in the visceral fat tissue. In parallel, parameters related to oxidative stress defence were also modulated, supporting the above-mentioned antioxidant activity [98]. Furthermore, in rats, a ginkgo extract improved the ovariectomy-induced resistance to serotonin-mediated hypophagia, at least in part through stimulation of the hypothalamic serotonergic activity [99]. The mechanisms by which ginkgo extract or its specific components may modulate energy homeostasis via the serotonergic system are only partly understood [100,101,102]. However, the induction of hypothalamic anorexigenic pro-opiomelanocortin (POMC), cocaine- and amphetamine-regulated transcript (CART), and 5-HT2C receptor gene expression, but not of orexigenic effectors in rats after a single ginkgo extract dose may suggest that components or specific compositions of this herb can act as rapid hypophagic appetite modulators [103].

These still small-scale preclinical results indicate that ginkgo extracts or isolated components may be biologically active in the regulation of metabolic and nutritional processes under certain conditions. However, this has to be further verified by systematic hypothesis-led and well-designed studies (model and disease conditions, compound compositions, dose levels, treatment regimes, and duration) in preclinical and clinical trials.

Advantageously, ginkgo extracts seem to be safe and well tolerated when moderate amounts, up to 240 mg per day, are ingested by humans [104]. The most commonly reported side effects are dizziness, gastrointestinal symptoms, and headache, but drug interactions with antithrombotic and non-steroidal anti-inflammatory drugs like clopidogrel and acetylsalicylic acid bear a minor bleeding risk [105].

## 4. *Bupleurum chinense*

Although little known outside Asia, the genus *Bupleurum*, especially the root of *B. chinense* (Radix bupleuri, Chai Hu) and that of many other subspecies, has been used for centuries for treatment of depression and other psychiatric disorders in China, Japan, South Korea, and other Asian countries. In traditional Chinese medicine (TCM), various formulas of Bupleurum in combination with other Chinese herbs, such as Chaihu–Shugan–San (CSS) and Xiao–Yao–San (XYS), have been widely applied for treatment of depression [106]. The China Food and Drug Administration (CFDA) has approved several of them [107]. However, no clinical studies using Radix bupleurum for mental health are listed in PubMed so far. Apart from this fact, few meta-analyses reported that herbal formulas containing *B. chinense*, either in combination with antidepressants or alone, can improve depressive symptoms and have low adverse effects. The remission rates were reported to be comparable or even higher than those achieved when antidepressants were used alone [108,109]. However, the robustness of the data is low. As rated by the authors themselves, the analysed studies were of high heterogeneity and low quality, not free from bias and partly without description of serious adverse events.

Despite this poor data situation, *B. chinense* is of interest in the search for drugs with a broad spectrum of activity contributing to mental health. As with other traditional herbal products, certain effects of Bupleurum-based formulations are attributed to a variety of bioactive ingredients. These mainly comprise terpenoids (triterpenoid and sterol saponins), phenols like flavonoids, phenylpropanoids with phenylpropionic acids (mainly caffeic acid) and lignans, mono- and sesquiterpens (essential oils), and many others. Many of these compounds have been shown to exert numerous synergistic pharmacological effects that could potentially be utilised in clinical applications [110].

Major components of the Bupleurum species are about 35 saponins, in particular saikosaponins A, C, and D (SSA, SSC, SSD), representing a group of glycosylated triterpenic oleanane and ursane derivatives. Preferably SSA, but also SSD, are considered as the main anti-inflammatory constituents of Bupleurum suppressing pro-inflammatory cytokines and associated proteins and thereby restricting the NF-κB pathway shown in vitro [111]. In addition, the inhibition of the mitogen-activated protein kinase (MAPK) pathway via reduction in phosphorylation of p38 MAPK, c-Jun *N*-terminal kinases (c-JNK), and extracellular signal-regulated kinases (ERK1/2) was repeatedly reported in vitro for both SSA and SSD [110]. Similarly, flavonoid aglycones like kaempferol and quercetin, preferentially abundant in plant’s overground parts, exert anti-inflammatory activity in response to the bacterial toxin LPS, but have also a powerful antioxidative capacity by free radical scavenging in kinetic-based structure–activity analysis [112].

It should be noted that secondary plant substances, among them polyphenols, have restricted access to the brain [113]. Likewise, blood-to-brain passage is very different for triterpenoid saponins [114]. Therefore, and in view of manifold conceivable derivatives produced by microbiota, intestine, or liver, the origin of pharmacological effects on brain health is mostly unclear [115]. Irrespective of that, preparations of Bupleurum herb combinations are integral parts of traditional medicine, obviously unintentionally utilising a synergistic multi-target treatment for depression to enhance anti-inflammatory effects and neuroprotection and to keep side effects low. Therefore, preclinical research is aimed at scientific evidence for effectiveness of such treatments and their underlying mechanisms.

An example for such preclinical investigations is Chaihu-Shugan-San (CSS), composed of Bupleurum and six other herbs. CSS was fed for the last two weeks of four-week period of unpredictable mild stress to rats [116]. CSS improved body weight gain and food intake and reduced depression-like behaviour in rats. Interestingly, when CSS was decomposed to its two individual components, Shu Gan and Rou Gan, containing 4 or 3 herbs, respectively, and fed in the model, only the Shu Gan component exerted antidepressant effects. Proteomics comparison of rat’s hippocampi revealed that complete CSS also altered the expression of proteins that were not regulated by these two components alone and vice versa. Further bioinformatics and protein analysis suggest that CSS modulated mainly antidepressant neurotransmitter pathways, most significant of GABA. Currently, there is no explanation for these complex interactions in target regulation by herb compositions.

In a similar preclinical chronic stress model in rats, one of the most accepted herbal pair formulations in TCM for antidepressant treatment, Chaihu–Baishao (radices of *B. chinense* and *Paeoniae lactiflora*) evoked superior synergistic antidepressant-like and anti-inflammatory activities compared to Bupleurum alone [117]. By subsequent network pharmacology with compound filtering, both herbs shared only one active compound out of 29. Furthermore, an average number of about 41 targets per compound and 6 compounds per target were calculated for the combination. From all identified targets, the MAPK pathway, as well as arachidonic acid and tryptophan metabolism, were most apparent, suggesting that this herb pair exerts combinatory effects on multiple depression-related signalling pathways in a comparable manner.

One reason for the improved effects of the herb combination of radices of Bupleurum and Paeoniae may be the enhanced bioavailability of several components including SSA, SSB2, and saikogenin derivatives in rats compared to administration of Bupleurum alone [118]. These data strongly argue for the importance of pharmacokinetic interaction of substance compositions, which subsequently may determine observable drug effects. Analogously, in an ex vivo rat model, SSA and SSD were poorly absorbed themselves, but they enhanced the intestinal wall passage of one of the most powerful antioxidant and anti-inflammatory active monoterpene glycosides of radix Paeoniae, paeoniflorin [119,120]. This is explained by the surfactant activity inherent in saponins that may increase the intestinal permeability by opening epithelial tight junctions. In addition, P-glycoprotein-mediated efflux and hydrolysis via glucosidases may interfere with the oral bioavailability of the manifold compounds in herb mixtures.

Furthermore, improved bioavailability of phytochemicals, when administered in combinations, was positively correlated with the appearance of their metabolites. The metabolites include picolinic acid, a by-product of the kynurenine pathway which is down-regulated in depression; itaconic acid, which inhibits the NLR family pyrin domain containing 3 (NLRP3) protein; and α-linolenic acid. These metabolites may substantiate the antidepressant properties of herbal combinations through a variety of synergistic neuroprotective, immunological, and anti-proliferative effects, as described in preclinical studies [118].

It is worth noting that radix Paeoniae reversed the dose-limiting hepatotoxicity of Bupleurum in rats, mediated by saikosaponin-induced inhibition of glutathione synthetase (GSS), subsequent oxidative stress, and activation of NF-κB/NLRP3 pathway. The chronic co-administration of both drugs not only evoked antidepressant-like effects in stressed rats, but it also may re-shape the hepatic redox balance, and inhibit inflammatory responses, possibly through the regulation of gut microbiota in favour of glucosidase activity to promote the conversion of saikosaponins into metabolites (saikogenins) without disturbing GSS-inhibiting properties [121].

Currently, modes of action of some individual Bupleurum active ingredients, prodrugs, or regulated endogenous metabolites are under preclinical investigation to further uncover their therapeutic potential [122]. Apart from antidepressant-like effects of Bupleurum extracts in stressed rats, components derived from their blood, like metabolites of SSA, SSB1, SSB2, SSC, and SSD; valerenic acid; glycyrrhetinic acid (enoloxone); and nootkatone individually improved reserpine-induced depression-like behaviour in the zebrafish. This behavioural adjustment was associated with increased 5-HT and lowered cortisol content [122]. Reproducible antidepressant-like effects of SSD in stressed rats were similarly associated with a reconstitution of plasma corticosterone and glucocorticoid receptor expression in the hippocampus [123]. These data suggest an SSD-mediated correction of the hypothalamic–pituitary–adrenal (HPA) axis dysfunction. Additionally, SSD improved hippocampal neurogenesis accompanied by a raise of phosphorylated cAMP response element-binding protein (p-CREB) and of BDNF levels, all essential for antidepressant-like effects.

The enhanced 5-HT in rodents and zebrafish after Bupleurum treatment may suggest that certain components might influence the regulation of appetite and weight, at least by stimulation of 5-HT2C receptors whose implication in depression is still under debate. In line with these findings, in structure–activity relationship studies, *B. chinense* extract and SSA itself exhibit strong 5-HT2C receptor agonistic activity [124]. In healthy rats, SSA elicited food intake suppression and weight gain inhibition, which is in agreement with the anorectic properties of 5-HT2C receptor agonists [124]. This raises the question of potential anti-obesity efficacy. This hypothesis is also supported by the dose-dependent effect of SSA to reduce the reinforcing and motivational properties of highly palatable, high caloric food or drugs and to suppress the cue-induced reinstatement of seeking behaviour, similar to the cannabinoid CB1 receptor antagonist/inverse agonist rimonabant, which was used as reference in this model [125,126]. The idea that SSA may have anorectic or anti-obesogenic effects should be pursued further using reproducible preclinical behavioural correlates by pharmacological intervention and with the observation of potential side effects.

It is likely that SSA may not only interfere with appetite-regulating neuronal mechanisms to suppress excessive food intake per se but also may dampen binge-like eating behaviour which also needs to be proven.

It is noteworthy that treatment of healthy mice for 34 days with a no further defined extract of *B. scorzonerifolium* Willd. by gavage did not affect the body weight gain [127]. The most likely explanation for this discrepancy is differences in the composition and content of saikosaponins in both plant subspecies. In particular, the amount of SSA is considerably higher in *B. chinense* [128,129]. This observation strongly points to the importance of well-defined standardised compositions and doses of natural products in terms of their target-orientated use in compromised individuals and evaluation of their potentially synergistic pharmacological basics.

## 5. *Berberis vulgaris*, *Coptis chinensis*, and Other Berberine-Containing Plants

Berberine (BBR) is one of the few alkaloids that are found not only in different genera of the same plant family, but also in plants of different families like *Berberidaceae* (*Berberis* spp.), *Ranunculaceae* (*Coptis* spp.), *Papaveraceae*, and *Caesalpineae*. Traditionally, plants containing BBR as a main active component, such as *Berberis vulgaris*, *Berberis aristata*, or *Coptis chinensis* (Huanglian), have been used to treat infections or diseases of skin or gastrointestinal tract. Nowadays, this isoquinoline alkaloid is also available by chemical synthesis.

Apart from current scientific interest in BBR to support glucose and lipid homeostasis and insulin sensitivity, it has become a social media hot-topic as a promoted dietary supplement with purported weight loss capabilities [130,131].

Indeed, a comprehensive systematic review and dose–response meta-analysis of clinical data found a significant reduction in body weight and body mass index (BMI) in adults by treatment with BBR [132]. Whereas BBR supplementation was mostly ineffective at normal BMI, in subgroups with impaired metabolic health, the effects of BBR on lipid profile, insulin resistance, and fasting glucose were significant, but required daily intake up to 1.8 g per day and at least a treatment duration of 40 weeks. The underlying data were reported to have a high risk of bias and inconsistent outcomes across individual trials. Therefore, there is still insufficient scientific evidence to conclude its clinical effectiveness.

In clinical practice, weight gain by psychotropic drugs with broad receptor profiles, e.g., the antipsychotic olanzapine or the antidepressant amitriptyline, is related to metabolic disorders and is one major reason for discontinuation of treatment [133]. A few preliminary clinical studies suggest that supplementation with BBR may alleviate this adverse effect. In one RCT, a small group of schizophrenic patients individually adjusted to one single antipsychotic was treated either with BBR (0.9 g daily, *n* = 27) or placebo (*n* = 22) [134]. Eight weeks after, the body weight was significantly lower in the BBR group, but limitations in study design, subject heterogeneity, and the lack of significance in the “before and after” within-group comparisons reduce the value of these data. In another RCT, mild metabolic alterations were induced by at least nine months of olanzapine treatment. Here, the BMI and other metabolic parameters were significantly improved after 12 weeks of accompanying treatment with BBR compared to placebo [135].

Preclinical studies revealed similar results. BBR (0.38 g/kg per day) and metformin (0.3 g/kg per day) were shown to prevent olanzapine-induced weight gain, white fat accumulation, and brown fat loss, responsible for thermogenesis in rats, but did not hinder the increased food intake [136]. This effect was explained by an increase in energy expenditure involving expression of the thermogenic AMP-activated protein kinase (AMPK) and uncoupling protein 3 (UCP3) genes, corresponding to reduced energy storage. Similarly, in mice, BBR and metformin lowered olanzapine-induced orexigenic signalling, obviously involving down-regulation of serum levels of ghrelin and leptin as well as reduced expression of orexigenic hypothalamic neuropeptides [137]. Interestingly, the olanzapine-induced hypothalamic expression of the transient receptor potential cation channels TRPV1/3, likely emerging targets in metabolic disorders, was attenuated by BBR.

Given that metformin may attenuate adverse metabolic effects of several psychotropic drugs and that common regulatory pathways are suggested for both, BBR may represent a justified alternative therapeutic strategy for mental health problems associated with metabolic or ED that needs to be systematically explored [133,138].

Patients with obesity often develop neuroinflammation accompanied by negative mood or depression, which is strongly associated with inadequate glucagon-like peptide-1 (GLP-1) signalling [139,140,141]. Therefore, direct or indirect stimulation of GLP-1 receptors is currently being discussed as a therapeutic option for BED, comorbid depression in obesity, or in psychopharmacological treatments associated with weight gain [142]. The latter has been demonstrated in four RCTs with weight loss outcomes following GLP-1 analogue treatment in patients with antipsychotic-induced weight gain [143,144].

It is therefore exciting that BBR increased the GLP-1 secretion in streptozotocin-induced diabetic rats and furthermore that antidiabetic-like effects (reduction in fasting glucose, increase in pancreatic insulin) and reduced food intake were observed in healthy rats after five weeks of 120 mg BBR/kg per day per os [145]. In a diabetic mouse model, BBR improved pancreatic islet β-cell function by activation of the GLP-1/GLP-1R/PKA signalling pathway in intestinal L-cells and in islet α-cells [146]. This item is further discussed below. Supporting these data, another animal study showed that BBR reduced HFD-induced weight gain in rats and suggested that BBR acts directly in the brain to counteract neuropeptide Y (NPY)-driven food intake [147].

While the appetite-suppressing effect of BBR may be appealing to patients with BED or with weight-related concerns, it could be dangerous when misused as it could exacerbate restrictive eating patterns or obsessive weight control behaviour. Therefore, the risk of its use in the context of such ED must be clarified.

On the other hand, although there is no robust evidence so far, the mild antidepressant and anxiolytic effects of BBR via serotonergic and dopaminergic pathways may improve mental health disturbances associated with ED. This is implied by preclinical studies in which BBR alleviates depressive-like behaviour induced by corticosterone administration or by chronic unpredictable mild stress (CUMS) in mice [148,149]. Improvements were related to anti-inflammatory processes, e.g., reduction in brain and peripheral cytokine levels and lower cerebral expression of NLRP3 inflammasome. Consistently, in two RCTs, BBR significantly improved negative symptoms and cognitive deficits in patients with schizophrenia [150,151]. These positive responses to BBR also appear to be associated with anti-inflammatory activity at the cytokine level.

In most studies, BBR was applied orally. However, its intestinal uptake is only approximate 0.5% due to its physicochemical properties, a high affinity to the efflux transporter P-glycoprotein and fast intestinal and hepatic first-pass metabolisation [152,153]. Consequently, mean achievable plasma concentrations of about 4 nM in animal models and humans, even after chronic administration, appear to be below the required concentrations for activation of AMPK for mitochondria function, for inhibition of acetylcholine esterase or for improvement of insulin resistance which all were reported to be in the µmolar range [154,155,156,157]. Pharmacokinetic studies in rats point to a ten-fold enrichment of BBR in liver compared to plasma, but only about 10% of plasma BBR seems to cross the blood–brain barrier (BBB) [152,153]. Furthermore, an improvement of viability of neuronal PC12 cells or of behaviour of 6-hydroxydopamine-treated zebrafish, both as models for neuroprotective effects of BBR, were observed only in concentrations above 0.1 µM [158].

All these uncertainties urgently call for systematic investigations of the relationship between BBR, its metabolites and supposed receptor binding affinities as well as consecutive target activities. Today, the manifold effects of BBR are only described at an observational level, but the molecular compound–target interactions necessary for these mechanisms remain largely unknown. Beyond that, questions arise as how such low plasma concentrations and limited access to the brain after oral BBR administration may mediate central effects on mental health in animal models and humans and whether such effects might also be attributable to the interaction of BBR with intestinal microbiome [159,160].

Apart from hepatic conversion into several active metabolites, gut microbiota reduces BBR to dihydroberberine, which has a five-fold higher absorption rate and is suggested to be re-oxidised in the intestine, thereby probably improving the parent compound bioavailability to the observed levels despite P-glycoprotein outward transport [157,161,162].

Not only the extent, but probably moreover the diversity of composition of the gut microbiome, is strongly related to psychiatric disorders such as anxiety and depression [163,164,165]. Simplified, even findings for alpha and beta diversity were inconsistent, higher abundance of pro-inflammatory species and lower short-chain fatty acid (SCFA) producing-bacteria seem to be related to intestinal, peripheral, and central inflammation.

In animal models of depression, BBR administration was shown to alter microbiota in favour of *Bacteroidetes* and *Lactobacilli* and *Bifidobacteria*, microbiota with anti-inflammatory and metabolic benefits, and to reduce *Firmicutes*. These changes were associated with an attenuation of gut-derived inflammation, the restoration of intestinal barrier integrity, and improvement of behavioural outcome [159,160,166]. SCFA, e.g., butyrate, propionate, and acetate, resulting from BBR-augmented carbohydrate fermentation increase the enteroendocrine activity, e.g., the release of GLP-1 and peptide YY from intestinal L cells as reviewed elsewhere [167]. Furthermore, they may enter the bloodstream as important signalling molecules within the gut–brain communication [166,168]. For example, SCFA affect the permeability of the blood–brain barrier and activate microglia [168]. Microbiota are also able to synthesise a range of major neurotransmitters such as biogenic amines, GABA or histamine. They may also alter the levels of neurotransmitters or other signalling molecules like peptides or steroids synthesised by the host. Thereby, the microbiome may communicate not only locally with the enteric nervous system but also contributes to the innervation of the vagus nerve or to the activity of the HPA axis [169]. Such a signalling might offer a plausible link between neuroinflammation, negative mood, and altered GLP-1 as outlined above.

Gut dysbiosis and reduced SCFA in faeces, intestine, and blood have repeatedly reported in ED, mainly for AN [170,171,172,173]. A strong case and progression sensitivity of microbiota alterations in AN was pointed out, which in future should be captured by longitudinal study designs considering clear diagnoses and self-reports. Most importantly, the composition of a “normal” microbiota, which differs dependent on geographic origin, eating habits, food choice, gender, age, and many other conditions, must be recognised to assess the effectiveness of clearly defined plant-derived products [174]. Even though data on the clinical use of BBR in ED and adjunctive therapies in AN are currently unavailable, the oral administration of BBR to address the restoration of intestinal microbiota and their fermentation products may be beneficial.

Having the low systemic bioavailability of BBR after oral administration in mind and looking for the missed junction to the observed effects of this compound, its interaction with gut microbiota and the restoration of their beneficial metabolic products such as SCFA or neurotransmitters may be an explanatory approach [175].

## 6. Summary and Future Implications

What important insights can be extracted from the above contemplations? Undoubtedly, there is some potential of the key ingredients discussed, particularly rosemary, Bupleurum, and Berberis, to positively influence physical and mental health.

For a large number of individual substances or extracts with different compositions, peripheral and in some cases also centrally mediated effects caused by interaction with certain pharmacological target structures have been demonstrated in vivo. Some of these effects could be of significance for the treatment of ED or body schema disorders associated with mental disorders.

Currently, favoured ingredients of plant products have primarily been investigated by mechanistic approaches in a fairly eclectic manner guided by traditional fields of application. Because of commonly used low or pharmaco-dynamically unclear doses of mixtures, no comprehensive dose–response profiles of specific substances in vivo and even less clear side-effect or toxicity profiles can be determined. Even though an often-favoured multi-target concept of low-dose substances with partly synergistic responses in the context of natural products may well have its justification, this does not necessarily lead to a conclusive theory of mechanisms of action. Thereby, several dose–effect relations remain unsolved which are mandatory for optimised use. Furthermore, studies with targeted combinations of biologically active compounds also have to scrutinise the relationship between synergistic efficacy and the resulting side effect profile.

As an important tool, the network pharmacology can help to complete the biological efficacy profile of a compound and to make synergistic principles more likely.

For effective exploitation of synergistic effects, however, the separate determination of the value and safety of the individual active ingredients in clearly defined pharmacodynamic study designs and dose–response relationships is essential.

The direct effects of rosemary’s main compounds in ED are imaginable, but currently not very substantiated and at best of subordinate importance. So, rosemary extracts have not yet been specifically investigated in ED. However, the effects of rosemary ingredients that strengthen mental health via various central neuronal mechanisms, e.g., serotonin turnover and/or GABA receptor modulation, have been documented in preclinical approaches. The broad anti-inflammatory and antioxidant activity of many individual rosemary ingredients in vitro, such as 1,8-cineole or carnosic acid, may further support central mechanisms. As the anti-inflammatory effect of 1,8-cineole is not limited to rosemary, but is attributed to many other plants too, it forms part of a respective overall effect.

Following oral administration in humans, favoured rosemary ingredients like carnosic acid, carnosol, and rosmanol/rosmarinic acid were found in plasma and the brain—a prerequisite for direct peripheral and central effects. In sum, the collected data suggest that a secondary influence on eating behaviour is expectable.

More confusing are the preclinical and clinical data for *Bupleurum chinensis* because, with a few exceptions, in animal models, predominantly traditionally prepared mixtures or parts of mixtures were used. Nonetheless, there is preclinical evidence that triterpenoid saponins and their intestinal derived metabolites, which have very different access to circulation and brain, can mediate measurable central effects in multiple depression-related signalling pathways alone but strengthened in combinations. However, no clear dose–response relationships are known. If the reported 5HT2C agonistic receptor activity of SSA in healthy animals can be further utilised to solely establish an anorectic principle applicable in ED or body schema disorders or whether a CB1 receptor antagonistic effect contributes still remains unclear. An essential step forward to exploiting the potential benefits of Bupleurum is the determination of the biological fate and bioavailability of pure lead compounds and their main metabolites according to established pharmacokinetic guidelines. Based on this, associated receptors and their signalling pathways should be investigated in systematic dose–response relationship studies. This also applies to the effects of SSD in terms of re-balancing the HPA axis function and hippocampal neurogenesis in stress-induced depression-like behavioural symptoms, mechanisms which are possibly also associated with ED.

In European medicine, the multi-ingredient mixture of *Gingko biloba* leaves enriched with ginkgolides and bilobalides, even when used in standardised extracts, has gained limited value in early types of dementia like AD and is only recommended as a ‘can’. Based on the current scientific literature, there is no evidence for an off-label medical use of ginkgo extracts in ED. Conceivable are only indirect effects on body weight due to modulation of central mechanisms involved in emotional regulation.

Forward-looking, this review refers to newer experimental data showing that a distinct ginkgo extract, when administered orally and subchronically to obese rats, reduced food intake, adiposity, and insulin resistance. Compatible with an induction of hypothalamic anorexigenic POMC, CART, and 5-HT2 gene expression in rats following one single dose of ginkgo extract only, components or compositions of this herb could potentially act as metabolic and hypophagic appetite modulators in future study approaches. However, these results must first be confirmed using defined extracts whose compositions have to be gradually minimised until individual key effects virtually disappear. Subsequent dose titrations with pure substances and recombination can provide further insight into the desired optimal effects.

For BBR, the review highlights some focal points of high interest for ED therapies. Firstly, preclinical and clinical studies, albeit with methodological weaknesses, indicate a beneficial effect comparable to metformin, but largely free of side effects that might be utilised in the future in antipsychotic-induced weight gain. The overall effect probably consists of an influence on fat thermogenesis, an increase in energy expenditure, and a reduction in orexigenic signalling. Secondly, in preclinical studies, BBR augments gastrointestinal carbohydrate fermentation associated with enhanced enteroendocrine activity. The GLP-1-like effects of BBR may be beneficial in pathological weight gain. However, given the low CNS levels that can be achieved, the direct contribution of BBR to central effects is questionable. Again, it follows that clarification of the pharmacokinetic fate of BBR in the human organism is an urgent task. Thirdly, gut dysbiosis is reported to be relevant in mental diseases as well as in ED, in particular in AN. As BBR is involved in the reorganisation of microbiome and its fermentation products, which probably serve as important systemic signalling molecules, it is worth being studied clinically in AN. Overall, BBR opens a potential alternative therapeutic tool for mental health problems associated with metabolic or eating disorders.

Inspired by traditional uses of important plants and/or their value-determining main ingredients, the present analysis of scientific literature outlines the currently known spectrum of their effects and potential benefits in ED bi-directionally linked to mental health problems. However, the highly heterogeneous features of traditional extracts without standardised phytochemical profiling limit generalised statements on health effects in human.

Successful research strategies can only generate valid efficacy data provided that mixtures of plant substances are standardised to proposed marker compounds or separated into their individual components, which makes it possible to recognise and to verify the contribution of each individual substance and its targets. This paves the way to effectively utilise a multi-target concept in later research phases and, in the best case, to incorporate it into the development of an approved drug.

In parallel, it is essential to continue pharmacological research with pure substances or at least with very clearly standardised downscaled extracts on carefully predefined targets and downstream pharmacodynamic mechanisms. Based on a hypothesised mechanism of action, future clinical trials need to verify the positive effects in body schema disorders. This requires the determination of dose–response relationships of well-defined compounds, strict inclusion and exclusion criteria for study participants, and valid primary outcome measures, arranged at best in longitudinal study designs, all preconditions that are often neglected in investigations of natural products.

At this point, it must be emphasised that the absolute prerequisite for clinical studies is the clarification of the biological fate and pharmacokinetic behaviour of the parent substance and the most important metabolites in order to assign effects and dose–response relationships reliably. The often-limited availability of such data inevitably impedes the uncovering of their pharmacological effects. A challenge in this context is the quantitative and qualitative variability of metabolites produced in the intestine and liver. Therefore, isolated active metabolites or chemically synthesised analogues are well suited to prove their access to brain and their peripheral and central pharmacodynamics.

Contrary to the mostly only observational investigations of compound- or composition-induced phenomenological responses, research must also rely on hypotheses on relevant single or multiple target structures. The further exploration and validation of such hypotheses should include network pharmacology modelling as well as metabolomics-integrated pharmacokinetics to strengthen proposed mechanistic explanations and to identify candidate lead compounds. This could be another starting point for further structure–activity relationship studies. In preclinical-to-clinical translation, compounds identified in this way will add value when applied in experimental models, e.g., tissue cultures, blood–brain barrier systems, or relevant behavioural phenotypes up to patient-derived organoids.

An alternative expedient research strategy draws upon a clear pharmacological target structure and screens natural products, which are characterised and covered in drug banks for target efficacy, in in vitro assays, as successfully demonstrated by Tarrago et al. (2007) for the prolyl oligopeptidase found to be inhibited by BBR [176]. Here, the traditional fields of application may also guide the selection process of compounds, supported by defined algorithms. In addition, such an approach also may promote biology-orientated compound synthesis based on the plant-derived parent substance.

In those approaches, the traditional application may also determine the process for compound selection at best supported by algorithms of artificial intelligence.

Despite methodical shortcomings, there is growing experimental evidence for pharmacologically comprehensible and therapeutically useful effects of natural compounds or mixtures in mental illness, associated ED, or body schema disorders.

## References

[1] Polivy J., Herman C.P. (2005). Mental health and eating behaviours: A bi-directional relation. Can. J. Public Health.

[2] Tan E.J., Raut T., Le L.K.-D., Hay P., Ananthapavan J., Lee Y.Y., Mihalopoulos C. (2023). The Association between Eating Disorders and Mental Health: An Umbrella Review. J. Eat. Disord..

[3] Borges R.S., Ortiz B.L.S., Pereira A.C.M., Keita H., Carvalho J.C.T. (2019). *Rosmarinus officinalis* Essential Oil: A Review of Its Phytochemistry, Anti-Inflammatory Activity, and Mechanisms of Action Involved. J. Ethnopharmacol..

[4] Quílez M., Ferreres F., López-Miranda S., Salazar E., Jordán M.J. (2020). Seed Oil from Mediterranean Aromatic and Medicinal Plants of the Lamiaceae Family as a Source of Bioactive Components with Nutritional. Antioxidants.

[5] Kitts D.D., Singh A., Fathordoobady F., Doi B., Pratap Singh A. (2019). Plant Extracts Inhibit the Formation of Hydroperoxides and Help Maintain Vitamin E Levels and Omega-3 Fatty Acids During High Temperature Processing and Storage of Hempseed and Soybean Oils. J. Food Sci..

[6] Nieto G., Ros G., Castillo J. (2018). Antioxidant and Antimicrobial Properties of Rosemary (*Rosmarinus officinalis*, L.): A Review. Medicines.

[7] (2012). Essential Oil of Rosemary (*Rosmarinus officinalis* L.). https://www.iso.org/standard/56521.html#lifecycle.

[8] Silva-Filho S., De Souza Silva-Comar F., Wiirzler L., Do Pinho R., Grespan R., Bersani-Amado C., Cuman R. (2015). Effect of Camphor on the Behavior of Leukocytes In Vitro and In Vivo in Acute Inflammatory Response. Trop. J. Pharm. Res..

[9] Nam S.-Y., Chung C., Seo J.-H., Rah S.-Y., Kim H.-M., Jeong H.-J. (2014). The Therapeutic Efficacy of α-Pinene in an Experimental Mouse Model of Allergic Rhinitis. Int. Immunopharmacol..

[10] Juhás Š., Bukovská A., Čikoš Š., Czikková S., Fabian D., Koppel J. (2009). Anti-Inflammatory Effects of *Rosmarinus officinalis* Essential Oil in Mice. Acta Vet. Brno.

[11] Vallverdú-Queralt A., Regueiro J., Martínez-Huélamo M., Rinaldi Alvarenga J.F., Leal L.N., Lamuela-Raventos R.M. (2014). A Comprehensive Study on the Phenolic Profile of Widely Used Culinary Herbs and Spices: Rosemary, Thyme, Oregano, Cinnamon, Cumin and Bay. Food Chem..

[12] Del Baño M.J., Lorente J., Castillo J., Benavente-García O., Del Río J.A., Ortuño A., Quirin K.-W., Gerard D. (2003). Phenolic Diterpenes, Flavones, and Rosmarinic Acid Distribution during the Development of Leaves, Flowers, Stems, and Roots of *Rosmarinus officinalis*. Antioxidant Activity. J. Agric. Food Chem..

[13] National Center for Biotechnology Information (2025). PubChem Compound Summary for CID 5281792, Rosmarinic Acid. https://pubchem.ncbi.nlm.nih.gov/compound/Rosmarinic-acid.

[14] Moreno S., Scheyer T., Romano C.S., Vojnov A.A. (2006). Antioxidant and Antimicrobial Activities of Rosemary Extracts Linked to Their Polyphenol Composition. Free Radic. Res..

[15] De Oliveira J.R., Camargo S.E.A., De Oliveira L.D. (2019). *Rosmarinus officinalis* L. (Rosemary) as Therapeutic and Prophylactic Agent. J. Biomed. Sci..

[16] Suganami T., Tanaka M., Ogawa Y. (2012). Adipose Tissue Inflammation and Ectopic Lipid Accumulation. Endocr. J..

[17] Dani C., Tarchi L., Cassioli E., Rossi E., Merola G.P., Ficola A., Cordasco V.Z., Ricca V., Castellini G. (2024). A Transdiagnostic and Diagnostic-Specific Approach on Inflammatory Biomarkers in Eating Disorders: A Meta-Analysis and Systematic Review. Psychiatry Res..

[18] Jiao W., Lin J., Deng Y., Ji Y., Liang C., Wei S., Jing X., Yan F. (2025). The Immunological Perspective of Major Depressive Disorder: Unveiling the Interactions between Central and Peripheral Immune Mechanisms. J. Neuroinflamm..

[19] Vallée A. (2022). Neuroinflammation in Schizophrenia: The Key Role of the WNT/β-Catenin Pathway. Int. J. Mol. Sci..

[20] Ercan P., El S.N. (2018). Bioaccessibility and inhibitory effects on digestive enzymes of carnosic acid in sage and rosemary. Int. J. Biol. Macromol..

[21] Romo Vaquero M., Yáñez-Gascón M.-J., García Villalba R., Larrosa M., Fromentin E., Ibarra A., Roller M., Tomás-Barberán F., Espín De Gea J.C., García-Conesa M.-T. (2012). Inhibition of Gastric Lipase as a Mechanism for Body Weight and Plasma Lipids Reduction in Zucker Rats Fed a Rosemary Extract Rich in Carnosic Acid. PLoS ONE.

[22] Liu Y., Zhang Y., Hu M., Li Y., Cao X. (2019). Carnosic Acid Alleviates Brain Injury through NF κB regulated Inflammation and Caspase 3 associated Apoptosis in High Fat induced Mouse Models. Mol. Med. Rep..

[23] Terzo S., Calvi P., Nuzzo D., Picone P., Allegra M., Mulè F., Amato A. (2023). Long-Term Ingestion of Sicilian Black Bee Chestnut Honey and/or D-Limonene Counteracts Brain Damage Induced by High Fat-Diet in Obese Mice. Int. J. Mol. Sci..

[24] Akbari S., Sohouli M.H., Ebrahimzadeh S., Ghanaei F.M., Hosseini A.F., Aryaeian N. (2022). Effect of Rosemary Leaf Powder with Weight Loss Diet on Lipid Profile, Glycemic Status, and Liver Enzymes in Patients with Nonalcoholic Fatty Liver Disease: A Randomized, Double-blind Clinical Trial. Phytother. Res..

[25] Oh E.S., Petersen K.S., Kris-Etherton P.M., Rogers C.J. (2020). Spices in a High-Saturated-Fat, High-Carbohydrate Meal Reduce Postprandial Proinflammatory Cytokine Secretion in Men with Overweight or Obesity: A 3-Period, Crossover, Randomized Controlled Trial. J. Nutr..

[26] Mourya A., Akhtar A., Ahuja S., Sah S.P., Kumar A. (2018). Synergistic Action of Ursolic Acid and Metformin in Experimental Model of Insulin Resistance and Related Behavioral Alterations. Eur. J. Pharmacol..

[27] Checker R., Sandur S.K., Sharma D., Patwardhan R.S., Jayakumar S., Kohli V., Sethi G., Aggarwal B.B., Sainis K.B. (2012). Potent Anti-Inflammatory Activity of Ursolic Acid, a Triterpenoid Antioxidant, Is Mediated through Suppression of NF-κB, AP-1 and NF-AT. PLoS ONE.

[28] Mirza F.J., Amber S., Sumera, Hassan D., Ahmed T., Zahid S. (2021). Rosmarinic Acid and Ursolic Acid Alleviate Deficits in Cognition, Synaptic Regulation and Adult Hippocampal Neurogenesis in an Aβ1-42-Induced Mouse Model of Alzheimer’s Disease. Phytomedicine.

[29] Hussain S.M., Syeda A.F., Alshammari M., Alnasser S., Alenzi N.D., Alanazi S.T., Nandakumar K. (2022). Cognition Enhancing Effect of Rosemary (*Rosmarinus officinalis* L.) in Lab Animal Studies: A Systematic Review and Meta-Analysis. Braz. J. Med. Biol. Res..

[30] Abdelhalim A., Karim N., Chebib M., Aburjai T., Khan I., Johnston G.A.R., Hanrahan J. (2015). Antidepressant, Anxiolytic and Antinociceptive Activities of Constituents from *Rosmarinus officinalis*. J. Pharm. Pharm. Sci..

[31] Juergens U.R., Stöber M., Vetter H. (1998). Inhibition of Cytokine Production and Arachidonic Acid Metabolism by Eucalyptol (1.8-Cineole) in Human Blood Monocytes in Vitro. Eur. J. Med. Res..

[32] Takaki I., Bersani-Amado L.E., Vendruscolo A., Sartoretto S.M., Diniz S.P., Bersani-Amado C.A., Cuman R.K.N. (2008). Anti-Inflammatory and Antinociceptive Effects of *Rosmarinus officinalis* L. Essential Oil in Experimental Animal Models. J. Med. Food.

[33] Zhang Q., Lenardo M.J., Baltimore D. (2017). 30 Years of NF-κB: A Blossoming of Relevance to Human Pathobiology. Cell.

[34] Guo Q., Jin Y., Chen X., Ye X., Shen X., Lin M., Zeng C., Zhou T., Zhang J. (2024). NF-κB in Biology and Targeted Therapy: New Insights and Translational Implications. Signal Transduct. Target. Ther..

[35] Pries R., Jeschke S., Leichtle A., Bruchhage K.-L. (2023). Modes of Action of 1,8-Cineol in Infections and Inflammation. Metabolites.

[36] Wu W., Wang D., Shi Y., Wang Y., Wu Y., Wu X., Shah B.A., Ye G. (2025). 1,8-Cineole Alleviates Hippocampal Oxidative Stress in CUMS Mice via the PI3K/Akt/Nrf2 Pathway. Nutrients.

[37] Machado D.G., Bettio L.E.B., Cunha M.P., Capra J.C., Dalmarco J.B., Pizzolatti M.G., Rodrigues A.L.S. (2009). Antidepressant-like Effect of the Extract of *Rosmarinus officinalis* in Mice: Involvement of the Monoaminergic System. Prog. Neuropsychopharmacol. Biol. Psychiatry.

[38] Machado D.G., Neis V.B., Balen G.O., Colla A., Cunha M.P., Dalmarco J.B., Pizzolatti M.G., Prediger R.D., Rodrigues A.L.S. (2012). Antidepressant-like Effect of Ursolic Acid Isolated from *Rosmarinus officinalis* L. in Mice: Evidence for the Involvement of the Dopaminergic System. Pharmacol. Biochem. Behav..

[39] Machado D.G., Cunha M.P., Neis V.B., Balen G.O., Colla A., Bettio L.E.B., Oliveira Á., Pazini F.L., Dalmarco J.B., Simionatto E.L. (2013). Antidepressant-like Effects of Fractions, Essential Oil, Carnosol and Betulinic Acid Isolated from *Rosmarinus officinalis* L.. Food Chem..

[40] Takeda H., Tsuji M., Inazu M., Egashira T., Matsumiya T. (2002). Rosmarinic Acid and Caffeic Acid Produce Antidepressive-like Effect in the Forced Swimming Test in Mice. Eur. J. Pharmacol..

[41] Lin S.-H., Chou M.-L., Chen W.-C., Lai Y.-S., Lu K.-H., Hao C.-W., Sheen L.-Y. (2015). A Medicinal Herb, *Melissa officinalis* L. Ameliorates Depressive-like Behavior of Rats in the Forced Swimming Test via Regulating the Serotonergic Neurotransmitter. J. Ethnopharmacol..

[42] Takeda H., Tsuji M., Miyamoto J., Matsumiya T. (2002). Rosmarinic Acid and Caffeic Acid Reduce the Defensive Freezing Behavior of Mice Exposed to Conditioned Fear Stress. Psychopharmacology.

[43] Tian G., Bartas K., Hui M., Chen L., Vasquez J.J., Azouz G., Derdeyn P., Manville R.W., Ho E.L., Fang A.S. (2024). Molecular and Circuit Determinants in the Globus Pallidus Mediating Control of Cocaine-Induced Behavioral Plasticity. Neuron.

[44] Sasaki K., Ferdousi F., Fukumitsu S., Kuwata H., Isoda H. (2021). Antidepressant- and Anxiolytic-like Activities of *Rosmarinus officinalis* Extract in Rodent Models: Involvement of Oxytocinergic System. Biomed. Pharmacother..

[45] Onaka T., Takayanagi Y. (2019). Role of Oxytocin in the Control of Stress and Food Intake. J. Neuroendocrinol..

[46] Nematolahi P., Mehrabani M., Karami-Mohajeri S., Dabaghzadeh F. (2018). Effects of *Rosmarinus officinalis* L. on Memory Performance, Anxiety, Depression, and Sleep Quality in University Students: A Randomized Clinical Trial. Complement. Ther. Clin. Pract..

[47] Araki R., Sasaki K., Onda H., Nakamura S., Kassai M., Kaneko T., Isoda H., Hashimoto K. (2020). Effects of Continuous Intake of Rosemary Extracts on Mental Health in Working Generation Healthy Japanese Men: Post-Hoc Testing of a Randomized Controlled Trial. Nutrients.

[48] Achour M., Ben Salem I., Ferdousi F., Nouira M., Ben Fredj M., Mtiraoui A., Isoda H., Saguem S. (2022). Rosemary Tea Consumption Alters Peripheral Anxiety and Depression Biomarkers: A Pilot Study in Limited Healthy Volunteers. J. Am. Nutr. Assoc..

[49] Casarotto P.C., Girych M., Fred S.M., Kovaleva V., Moliner R., Enkavi G., Biojone C., Cannarozzo C., Sahu M.P., Kaurinkoski K. (2021). Antidepressant Drugs Act by Directly Binding to TRKB Neurotrophin Receptors. Cell.

[50] Azizi S., Mohamadi N., Sharififar F., Dehghannoudeh G., Jahanbakhsh F., Dabaghzadeh F. (2022). Rosemary as an Adjunctive Treatment in Patients with Major Depressive Disorder: A Randomized, Double-blind, Placebo-controlled Trial. Complement. Ther. Clin. Pract..

[51] Schneider E., Schmidt R., Cryan J.F., Hilbert A. (2024). A Role for the Microbiota-Gut-Brain Axis in Avoidant/Restrictive Food Intake Disorder: A New Conceptual Model. Int. J. Eat. Disord..

[52] Helal P., Xia W., Sardar P., Conway-Morris A., Conway-Morris A., Pedicord V.A., Serfontein J. (2024). Changes in the Firmicutes to Bacteriodetes Ratio in the Gut Microbiome in Individuals with Anorexia Nervosa Following Inpatient Treatment: A Systematic Review and a Case Series. Brain Behav..

[53] Romeo M., Cavaliere G., Traina G. (2024). Bulimia Nervosa and Depression, from the Brain to the Gut Microbiota and Back. Front. Biosci..

[54] Guo W., Xiong W. (2024). From Gut Microbiota to Brain: Implications on Binge Eating Disorders. Gut Microbes.

[55] Winter G., Hart R.A., Charlesworth R.P.G., Sharpley C.F. (2018). Gut Microbiome and Depression: What We Know and What We Need to Know. Rev. Neurosci..

[56] Bogielski B., Michalczyk K., Głodek P., Tempka B., Gębski W., Stygar D. (2024). Association between Small Intestine Bacterial Overgrowth and Psychiatric Disorders. Front. Biosci..

[57] Pisanu C., Squassina A. (2018). We Are Not Alone in Our Body: Insights into the Involvement of Microbiota in the Etiopathogenesis and Pharmacology of Mental Illness. Curr. Drug Metab..

[58] Zheng P., Zeng B., Zhou C., Liu M., Fang Z., Xu X., Zeng L., Chen J., Fan S., Du X. (2016). Gut Microbiome Remodeling Induces Depressive-like Behaviors through a Pathway Mediated by the Host’s Metabolism. Mol. Psychiatry.

[59] Pferschy-Wenzig E.-M., Pausan M.R., Ardjomand-Woelkart K., Röck S., Ammar R.M., Kelber O., Moissl-Eichinger C., Bauer R. (2022). Medicinal Plants and Their Impact on the Gut Microbiome in Mental Health: A Systematic Review. Nutrients.

[60] Dinan K., Dinan T. (2022). Antibiotics and Mental Health: The Good, the Bad and the Ugly. J. Intern. Med..

[61] Endo T.H., Santos M.H.D.M., Scandorieiro S., Gonçalves B.C., Vespero E.C., Perugini M.R.E., Pavanelli W.R., Nakazato G., Kobayashi R.K.T. (2025). Selective Serotonin Reuptake Inhibitors: Antimicrobial Activity Against ESKAPEE Bacteria and Mechanisms of Action. Antibiotics.

[62] Macedo D., Filho A.J.M.C., Soares De Sousa C.N., Quevedo J., Barichello T., Júnior H.V.N., Freitas De Lucena D. (2017). Antidepressants, Antimicrobials or Both? Gut Microbiota Dysbiosis in Depression and Possible Implications of the Antimicrobial Effects of Antidepressant Drugs for Antidepressant Effectiveness. J. Affect. Disord..

[63] Guo Y., Xie J., Li X., Yuan Y., Zhang L., Hu W., Luo H., Yu H., Zhang R. (2018). Antidepressant Effects of Rosemary Extracts Associate With Anti-Inflammatory Effect and Rebalance of Gut Microbiota. Front. Pharmacol..

[64] Romo Vaquero M., García Villalba R., Larrosa M., Yáñez-Gascón M.J., Fromentin E., Flanagan J., Roller M., Tomás-Barberán F.A., Espín J.C., García-Conesa M. (2013). Bioavailability of the Major Bioactive Diterpenoids in a Rosemary Extract: Metabolic Profile in the Intestine, Liver, Plasma, and Brain of Zucker Rats. Mol. Nutr. Food Res..

[65] Ghasemzadeh Rahbardar M., Hosseinzadeh H. (2025). Toxicity and Safety of Rosemary (*Rosmarinus officinalis*): A Comprehensive Review. Naunyn-Schmiedeberg’s Arch. Pharmacol..

[66] Younes M., Aggett P., Aguilar F., Crebelli R., Dusemund B., Filipič M., Frutos M.J., Galtier P., Gott D., EFSA ANS Panel (EFSA Panel on Food Additives and Nutrient Sources added to Food) (2018). Scientific Opinion on the refined exposure assessment of extracts of rosemary (E 392) from its use as food additive. EFSA J..

[67] Phipps K.R., Lozon D., Baldwin N. (2021). Genotoxicity and Subchronic Toxicity Studies of Supercritical Carbon Dioxide and Acetone Extracts of Rosemary. Regul. Toxicol. Pharmacol..

[68] WHO (2017). Safety Evaluation of Certain Food Additives: Prepared by the Eighty-Second Meeting of the Joint FAO/WHO Expert Committee on Food Additives (JECFA).

[69] Phipps K.R., Danielewska-Nikiel B., Mushonganono J., Baldwin N. (2021). Reproductive and Developmental Toxicity Screening Study of an Acetone Extract of Rosemary. Regul. Toxicol. Pharmacol..

[70] Norwegian Institute of Public Health W.H.O. Collaborating Centre for Drug Statistics Methodology. Last Updated 27 December 2024. https://atcddd.fhi.no/atc_ddd_index/?code=N06DX02.

[71] European Union Herbal Monograph on *Ginkgo biloba* L., Folium EMA/HMPC/321097/2012. https://www.ema.europa.eu/en/documents/herbal-monograph/final-european-union-herbal-monograph-ginkgo-biloba-l-folium_en.pdf.

[72] Kulić Ž., Lehner M.D., Dietz G.P.H. (2022). *Ginkgo biloba* Leaf Extract EGb 761^®^ as a Paragon of the Product by Process Concept. Front. Pharmacol..

[73] Napryeyenko O., Sonnik G., Tartakovsky I. (2009). Efficacy and Tolerability of *Ginkgo biloba* Extract EGb 761^®^ by Type of Dementia: Analyses of a Randomised Controlled Trial. J. Neurol. Sci..

[74] Kanowski S., Hoerr R. (2003). *Ginkgo biloba* Extract EGb 761 in Dementia: Intent-to-Treat Analyses of a 24-Week, Multi-Center, Double-Blind, Placebo-Controlled, Randomized Trial. Pharmacopsychiatry.

[75] Vellas B., Coley N., Ousset P.-J., Berrut G., Dartigues J.-F., Dubois B., Grandjean H., Pasquier F., Piette F., Robert P. (2012). Long-Term Use of Standardised *Ginkgo biloba* Extract for the Prevention of Alzheimer’s Disease (GuidAge): A Randomised Placebo-Controlled Trial. Lancet Neurol..

[76] Snitz B.E., O’Meara E.S., Carlson M.C., Arnold A.M., Ives D.G., Rapp S.R., Saxton J., Lopez O.L., Dunn L.O., Sink K.M. (2009). *Ginkgo biloba* for Preventing Cognitive Decline in Older Adults: A Randomized Trial. JAMA.

[77] DeKosky S.T., Williamson J.D., Fitzpatrick A.L., Kronmal R.A., Ives D.G., Saxton J.A., Lopez O.L., Burke G., Carlson M.C., Fried L.P. (2008). *Ginkgo biloba* for Prevention of Dementia: A Randomized Controlled Trial. JAMA.

[78] Necula B.-R., Necula R.D., Petric P.S., Ifteni P.I., Irimie M., Dima L. (2024). EGb761 Trials for Mild-to-Moderate Dementia—What Have We Learned in the Past 18 Years?. Am. J. Ther..

[79] Birks J., Grimley Evans J. (2009). *Ginkgo biloba* for Cognitive Impairment and Dementia. Cochrane Database Syst. Rev..

[80] National Institutes of Health (NIH), National Center for Complementary and Integrative Health (NCCIH) Last Updated February 2025. https://www.nccih.nih.gov/health/ginkgo.

[81] Savaskan E., Mueller H., Hoerr R., Von Gunten A., Gauthier S. (2018). Treatment Effects of *Ginkgo biloba* Extract EGb 761^®^ on the Spectrum of Behavioral and Psychological Symptoms of Dementia: Meta-Analysis of Randomized Controlled Trials. Int. Psychogeriatr..

[82] DGN e. V. & DGPPN e. V. (Hrsg.) S3-Leitlinie Demenzen—Living Guideline, Version 5.1, 28 March 2025. https://register.awmf.org/de/leitlinien/detail/038-013.

[83] Liu L., Wang Y., Zhang J., Wang S. (2021). Advances in the Chemical Constituents and Chemical Analysis of *Ginkgo biloba* Leaf, Extract, and Phytopharmaceuticals. J. Pharm. Biomed. Anal..

[84] Le Bars P.L., Velasco F.M., Ferguson J.M., Dessain E.C., Kieser M., Hoerr R. (2002). Influence of the Severity of Cognitive Impairment on the Effect of the *Ginkgo biloba* Extract EGb 761^®^ in Alzheimer’s Disease. Neuropsychobiology.

[85] Sarris J. (2018). Herbal Medicines in the Treatment of Psychiatric Disorders: 10-year Updated Review. Phytother. Res..

[86] Nurzyńska-Wierdak R. (2024). Plants with Potential Importance in Supporting the Treatment of Depression: Current Trends, and Research. Pharmaceuticals.

[87] Grosso C., Santos M., Barroso M.F. (2023). From Plants to Psycho-Neurology: Unravelling the Therapeutic Benefits of Bioactive Compounds in Brain Disorders. Antioxidants.

[88] Lin J., Sun X., Yang L. (2024). Effects and Safety of *Ginkgo biloba* on Depression: A Systematic Review and Meta-Analysis. Front. Pharmacol..

[89] Biernacka P., Adamska I., Felisiak K. (2023). The Potential of *Ginkgo biloba* as a Source of Biologically Active Compounds—A Review of the Recent Literature and Patents. Molecules.

[90] Bogacz A., Karasiewicz M., Dziekan K., Procyk D., Górska-Paukszta M., Kowalska A., Mikołajczak P.Ł., Ożarowski M., Czerny B. (2016). Impact of Panax Ginseng and *Ginkgo biloba* Extracts on Expression Level of Transcriptional Factors and Xenobiotic-Metabolizing Cytochrome P450 Enzymes. Herba Pol..

[91] Suárez-González E., Sandoval-Ramírez J., Flores-Hernández J., Carrasco-Carballo A. (2023). *Ginkgo biloba*: Antioxidant Activity and In Silico Central Nervous System Potential. Curr. Issues Mol. Biol..

[92] Ayatollahi S.A., Khoshsirat S., Peyvandi A.A., Rezaei O., Mehrjardi F.Z., Nahavandi A., Niknazar S. (2020). *Ginkgo biloba* modulates hippocampal BDNF expression in a rat model of chronic restraint stress-induced depression. Physiol. Pharmacol..

[93] Yeh K.-Y., Shou S.-S., Lin Y.-X., Chen C.-C., Chiang C.-Y., Yeh C.-Y. (2015). Effect of *Ginkgo biloba* Extract on Lipopolysaccharide-Induced Anhedonic Depressive-like Behavior in Male Rats. Phytother. Res..

[94] Montes P., Ruiz-Sanchez E., Rojas C., Rojas P. (2015). *Ginkgo biloba* Extract 761: A Review of Basic Studies and Potential Clinical Use in Psychiatric Disorders. CNS Neurol. Disord. Drug Targets.

[95] Kuribara H., Weintraub S.T., Yoshihama T., Maruyama Y. (2003). An Anxiolytic-Like Effect of *Ginkgo biloba* Extract and Its Constituent, Ginkgolide-A, in Mice. J. Nat. Prod..

[96] Rodriguez De Turco E.B., Droy-Lefaix M.T., Bazan N.G. (1993). EGb 761 Inhibits Stress-Induced Polydipsia in Rats. Physiol. Behav..

[97] Banin R.M., Hirata B.K.S., Andrade I.S., Zemdegs J.C.S., Clemente A.P.G., Dornellas A.P.S., Boldarine V.T., Estadella D., Albuquerque K.T., Oyama L.M. (2014). Beneficial Effects of *Ginkgo biloba* Extract on Insulin Signaling Cascade, Dyslipidemia, and Body Adiposity of Diet-Induced Obese Rats. Braz. J. Med. Biol. Res..

[98] Hirata B.K.S., Pedroso A.P., Machado M.M.F., Neto N.I.P., Perestrelo B.O., De Sá R.D.C.C., Alonso-Vale M.I.C., Nogueira F.N., Oyama L.M., Ribeiro E.B. (2019). *Ginkgo biloba* Extract Modulates the Retroperitoneal Fat Depot Proteome and Reduces Oxidative Stress in Diet-Induced Obese Rats. Front. Pharmacol..

[99] Banin R.M., De Andrade I.S., Cerutti S.M., Oyama L.M., Telles M.M., Ribeiro E.B. (2017). *Ginkgo biloba* Extract (GbE) Stimulates the Hypothalamic Serotonergic System and Attenuates Obesity in Ovariectomized Rats. Front. Pharmacol..

[100] Huguet F., Drieu K., Piriou A. (1994). Decreased Cerebral 5-HT1A Receptors during Ageing: Reversal by *Ginkgo biloba* Extract (EGb 761). J. Pharm. Pharmacol..

[101] Bolaños-Jiménez F., De Castro R.M., Sarhan H., Prudhomme N., Drieu K., Fillion G. (1995). Stress-induced 5-HT1A Receptor Desensitization: Protective Effects of *Ginkgo biloba* Extract (EGb 761). Fundam. Clin. Pharmacol..

[102] Sloley B.D., Urichuk L.J., Morley P., Durkin J., Shan J.J., Pang P.K.T., Coutts R.T. (2000). Identification of Kaempferol as a Monoamine Oxidase Inhibitor and Potential Neuroprotectant in Extracts of *Ginkgo biloba* Leaves. J. Pharm. Pharmacol..

[103] Machado M.M.F., Pereira J.P., Hirata B.K.S., Júlio V.S., Banin R.M., Andrade H.M., Ribeiro E.B., Cerutti S.M., Telles M.M. (2021). A Single Dose of *Ginkgo biloba* Extract Induces Gene Expression of Hypothalamic Anorexigenic Effectors in Male Rats. Brain Sci..

[104] Unger M. (2013). Pharmacokinetic Drug Interactions Involving *Ginkgo biloba*. Drug Metab. Rev..

[105] Mai N.T.Q., Hieu N.V., Ngan T.T., Van Anh T., Van Linh P., Thu Phuong N.T. (2025). Impact of *Ginkgo biloba* Drug Interactions on Bleeding Risk and Coagulation Profiles: A Comprehensive Analysis. PLoS ONE.

[106] Zeng L., Cao Y., Wang L., Dai Y., Hu L., Wang Q., Zhu L., Bao W., Zou Y., Chen Y. (2017). Role of Medicinal Plants for Liver-Qi Regulation Adjuvant Therapy in Post-stroke Depression: A Systematic Review of Literature. Phytother. Res..

[107] National Medical Products Administration. https://english.nmpa.gov.cn/drugs.html.

[108] Wang Y., Fan R., Huang X. (2012). Meta-Analysis of the Clinical Effectiveness of Traditional Chinese Medicine Formula Chaihu-Shugan-San in Depression. J. Ethnopharmacol..

[109] Yang L., Shergis J.L., Di Y.M., Zhang A.L., Lu C., Guo X., Fang Z., Xue C.C., Li Y. (2020). Managing Depression with *Bupleurum chinense* Herbal Formula: A Systematic Review and Meta-Analysis of Randomized Controlled Trials. J. Altern. Complement. Med..

[110] Teng L., Guo X., Ma Y., Xu L., Wei J., Xiao P. (2023). A Comprehensive Review on Traditional and Modern Research of the Genus Bupleurum (*Bupleurum* L., *Apiaceae*) in Recent 10 Years. J. Ethnopharmacol..

[111] Zhu J., Luo C., Wang P., He Q., Zhou J., Peng H. (2013). Saikosaponin A Mediates the Inflammatory Response by Inhibiting the MAPK and NF-κB Pathways in LPS-Stimulated RAW 264.7 Cells. Exp. Ther. Med..

[112] Di Meo F., Lemaur V., Cornil J., Lazzaroni R., Duroux J.-L., Olivier Y., Trouillas P. (2013). Free Radical Scavenging by Natural Polyphenols: Atom versus Electron Transfer. J. Phys. Chem. A.

[113] Figueira I., Menezes R., Macedo D., Costa I., Dos Santos C.N. (2017). Polyphenols Beyond Barriers: A Glimpse into the Brain. Curr. Neuropharmacol..

[114] Stępnik K. (2021). Biomimetic Chromatographic Studies Combined with the Computational Approach to Investigate the Ability of Triterpenoid Saponins of Plant Origin to Cross the Blood–Brain Barrier. Int. J. Mol. Sci..

[115] Manach C., Scalbert A., Morand C., Rémésy C., Jiménez L. (2004). Polyphenols: Food Sources and Bioavailability. Am. J. Clin. Nutr..

[116] Zhu X., Li T., Hu E., Duan L., Zhang C., Wang Y., Tang T., Yang Z., Fan R. (2022). Proteomics Study Reveals the Anti-Depressive Mechanisms and the Compatibility Advantage of Chaihu-Shugan-San in a Rat Model of Chronic Unpredictable Mild Stress. Front. Pharmacol..

[117] Li X., Qin X.-M., Tian J.-S., Gao X.-X., Du G.-H., Zhou Y.-Z. (2021). Integrated Network Pharmacology and Metabolomics to Dissect the Combination Mechanisms of *Bupleurum chinense* DC-Paeonia Lactiflora Pall Herb Pair for Treating Depression. J. Ethnopharmacol..

[118] Chen C., Yin Q., Tian J., Gao X., Qin X., Du G., Zhou Y. (2021). Studies on the Changes of Pharmacokinetics Behaviors of Phytochemicals and the Influence on Endogenous Metabolites After the Combination of Radix Bupleuri and Radix Paeoniae Alba Based on Multi-Component Pharmacokinetics and Metabolomics. Front. Pharmacol..

[119] Chen Y., Wang J., Yuan L., Zhou L., Jia X., Tan X. (2011). Interaction of the Main Components from the Traditional Chinese Drug Pair Chaihu-Shaoyao Based on Rat Intestinal Absorption. Molecules.

[120] He D.-Y., Dai S.-M. (2011). Anti-Inflammatory and Immunomodulatory Effects of Paeonia Lactiflora Pall., a Traditional Chinese Herbal Medicine. Front. Pharmacol..

[121] Chen C., Gong W., Tian J., Gao X., Qin X., Du G., Zhou Y. (2023). Radix Paeoniae Alba Attenuates Radix Bupleuri-Induced Hepatotoxicity by Modulating Gut Microbiota to Alleviate the Inhibition of Saikosaponins on Glutathione Synthetase. J. Pharm. Anal..

[122] Wu S., Li H.-M., Bing Y.-F., Zheng Y., Li W.-L., Zou X., Qu Z.-Y. (2023). Bupleurum Scorzonerifolium: Systematic Research through Pharmacodynamics and Serum Pharmacochemistry on Screening Antidepressant Q-Markers for Quality Control. J. Pharm. Biomed. Anal..

[123] Li H.-Y., Zhao Y.-H., Zeng M.-J., Fang F., Li M., Qin T.-T., Ye L.-Y., Li H.-W., Qu R., Ma S.-P. (2017). Saikosaponin D Relieves Unpredictable Chronic Mild Stress Induced Depressive-like Behavior in Rats: Involvement of HPA Axis and Hippocampal Neurogenesis. Psychopharmacology.

[124] Sun C.-L., Geng C.-A., Huang X.-Y., Ma Y.-B., Zheng X.-H., Yang T.-H., Chen X.-L., Yin X.-J., Zhang X.-M., Chen J.-J. (2017). Bioassay-Guided Isolation of Saikosaponins with Agonistic Activity on 5-Hydroxytryptamine 2C Receptor from *Bupleurum chinense* and Their Potential Use for the Treatment of Obesity. Chin. J. Nat. Med..

[125] Maccioni P., Fara F., Gessa G.L., Carai M.A.M., Chin Y.-W., Lee J.H., Kwon H.C., Colombo G. (2018). Reducing Effect of Saikosaponin A, an Active Ingredient of Bupleurum Falcatum, on Intake of Highly Palatable Food in a Rat Model of Overeating. Front. Psychiatry.

[126] Maccioni P., Chin Y.-W., Corelli F., Kwon H.C., Colombo G. (2023). Reducing Effect of Intragastrically Administered Saikosaponin A on Alcohol and Sucrose Self-Administration in Rats. Nat. Prod. Res..

[127] Bi Y., Li M., Wang Y., Yao J., Wang Y., Wang S., Zhuang L., Liu S., Li Z., Hao Z. (2025). Saikosaponins from Bupleurum Scorzonerifolium Willd. Alleviates Microglial Pyroptosis in Depression by Binding and Inhibiting P2X7 Expression. Phytomedicine.

[128] Li M., Zhang Q., Zhu T., Liu G., Chen W., Chen Y., Bu X., Zhang Z., Zhang Y. (2025). Broadly Targeted Metabolomics Analysis of Differential Metabolites Between *Bupleurum chinense* DC. and Bupleurum Scorzonerifolium Willd. Metabolites.

[129] Wang Z., Tian L., Xiao Y., Zhao M., Chang Y., Zhou Y., Liu S., Zhao H., Xiu Y. (2023). Quantitative and Differential Analysis between *Bupleurum chinense* DC. and Bupleurum scorzonerifolium Willd. Using HPLC-MS and GC-MS Coupled with Multivariate Statistical Analysis. Molecules.

[130] National Institutes of Health (NIH), National Center for Complementary and Integrative Health (NCCIH) Last Updated June 2023. https://www.nccih.nih.gov/health/in-the-news-berberine.

[131] National Institutes of Health (NIH), National Center for Complementary and Integrative Health (NCCIH) Last Updated November 2023. https://www.nccih.nih.gov/health/berberine-and-weight-loss-what-you-need-to-know.

[132] Zamani M., Zarei M., Nikbaf-Shandiz M., Hosseini S., Shiraseb F., Asbaghi O. (2022). The Effects of Berberine Supplementation on Cardiovascular Risk Factors in Adults: A Systematic Review and Dose-Response Meta-Analysis. Front. Nutr..

[133] Miedlich S.U., Lamberti J.S. (2025). Connecting the Dots: Understanding and Addressing the Metabolic Impact of Antipsychotic and Antidepressant Medications. Ann. N. Y. Acad. Sci..

[134] Qiu Y., Li M., Zhang Y., Liu Y., Zhao Y., Zhang J., Jia Q., Li J. (2022). Berberine Treatment for Weight Gain in Patients with Schizophrenia by Regulating Leptin Rather than Adiponectin. Asian J. Psychiatry.

[135] Pu Z., Sun Y., Jiang H., Hou Q., Yan H., Wen H., Li G. (2021). Effects of Berberine on Gut Microbiota in Patients with Mild Metabolic Disorders Induced by Olanzapine. Am. J. Chin. Med..

[136] Hu Y., Young A.J., Ehli E.A., Nowotny D., Davies P.S., Droke E.A., Soundy T.J., Davies G.E. (2014). Metformin and Berberine Prevent Olanzapine-Induced Weight Gain in Rats. PLoS ONE.

[137] Singh R., Bansal Y., Sodhi R.K., Singh D.P., Bishnoi M., Kondepudi K.K., Medhi B., Kuhad A. (2020). Berberine Attenuated Olanzapine-Induced Metabolic Alterations in Mice: Targeting Transient Receptor Potential Vanilloid Type 1 and 3 Channels. Life Sci..

[138] Moss L., Laudenslager M., Steffen K.J., Sockalingam S., Coughlin J.W. (2025). Antidepressants and Weight Gain: An Update on the Evidence and Clinical Implications. Curr. Obes. Rep..

[139] Piątkowska-Chmiel I., Wicha-Komsta K., Pawłowski K., Syrytczyk A., Kocki T., Dudka J., Herbet M. (2025). Beyond Diabetes: Semaglutide’s Role in Modulating Mood Disorders through Neuroinflammation Pathways. Cell. Mol. Neurobiol..

[140] Yang K., Wu Y., He Y., Dai J., Luo Y., Xie J., Ding W. (2025). GLP-1 and IL-6 Regulates Obesity in the Gut and Brain. Life Sci..

[141] Shah M., Vella A. (2014). Effects of GLP-1 on Appetite and Weight. Rev. Endocr. Metab. Disord..

[142] Himmerich H., McElroy S.L. (2024). Glucagon-Like Peptide 1 Receptor Agonists in Psychiatry. J. Clin. Psychopharmacol..

[143] Trott M., Arnautovska U., Siskind D. (2024). GLP-1 Receptor Agonists and Weight Loss in Schizophrenia—Past, Present, and Future. Curr. Opin. Psychiatry.

[144] Chan M., Qin Z., Man S., Lam M., Lai W.H., Ng R.M.K., Lee C.K., Wong T.L., Lee E.H.M., Wong H.K. (2022). Adjunctive Berberine Reduces antipsychotic-associated Weight Gain and Metabolic Syndrome in Patients with Schizophrenia: A Randomized Controlled Trial. Psychiatry Clin. Neurosci..

[145] Yu Y., Liu L., Wang X., Liu X., Liu X., Xie L., Wang G. (2010). Modulation of Glucagon-like Peptide-1 Release by Berberine: In Vivo and in Vitro Studies. Biochem. Pharmacol..

[146] Wu W., Xia Q., Guo Y., Wang H., Dong H., Lu F., Yuan F. (2023). Berberine Enhances the Function of Db/Db Mice Islet β Cell through GLP-1/GLP-1R/PKA Signaling Pathway in Intestinal L Cell and Islet α Cell. Front. Pharmacol..

[147] Park H.-J., Jung E., Shim I. (2020). Berberine for Appetite Suppressant and Prevention of Obesity. Biomed. Res. Int..

[148] Qin Z., Shi D.-D., Li W., Cheng D., Zhang Y.-D., Zhang S., Tsoi B., Zhao J., Wang Z., Zhang Z.-J. (2023). Berberine Ameliorates Depression-like Behaviors in Mice via Inhibiting NLRP3 Inflammasome-Mediated Neuroinflammation and Preventing Neuroplasticity Disruption. J. Neuroinflammation.

[149] Ge P., Qu S., Ni S., Yao Z., Qi Y., Zhao X., Guo R., Yang N., Zhang Q., Zhu H. (2023). Berberine Ameliorates Depression-like Behavior in CUMS Mice by Activating TPH1 and Inhibiting IDO1 -associated with Tryptophan Metabolism. Phytother. Res..

[150] Pu Z., Wen H., Jiang H., Hou Q., Yan H. (2023). Berberine Improves Negative Symptoms and Cognitive Function in Patients with Chronic Schizophrenia via Anti-Inflammatory Effect: A Randomized Clinical Trial. Chin. Med..

[151] Li M., Qiu Y., Zhang J., Zhang Y., Liu Y., Zhao Y., Jia Q., Fan X., Li J. (2022). Improvement of Adjunctive Berberine Treatment on Negative Symptoms in Patients with Schizophrenia. Eur. Arch. Psychiatry Clin. Neurosci..

[152] Liu Y.-T., Hao H.-P., Xie H.-G., Lai L., Wang Q., Liu C.-X., Wang G.-J. (2010). Extensive Intestinal First-Pass Elimination and Predominant Hepatic Distribution of Berberine Explain Its Low Plasma Levels in Rats. Drug Metab. Dispos..

[153] Tan X.-S., Ma J.-Y., Feng R., Ma C., Chen W.-J., Sun Y.-P., Fu J., Huang M., He C.-Y., Shou J.-W. (2013). Tissue Distribution of Berberine and Its Metabolites after Oral Administration in Rats. PLoS ONE.

[154] Chen W., Miao Y.-Q., Fan D.-J., Yang S.-S., Lin X., Meng L.-K., Tang X. (2011). Bioavailability Study of Berberine and the Enhancing Effects of TPGS on Intestinal Absorption in Rats. AAPS PharmSciTech.

[155] Liu C.-S., Zheng Y.-R., Zhang Y.-F., Long X.-Y. (2016). Research Progress on Berberine with a Special Focus on Its Oral Bioavailability. Fitoterapia.

[156] Ji H.-F., Shen L. (2012). Molecular Basis of Inhibitory Activities of Berberine against Pathogenic Enzymes in Alzheimer’s Disease. Sci. World J..

[157] Spinozzi S., Colliva C., Camborata C., Roberti M., Ianni C., Neri F., Calvarese C., Lisotti A., Mazzella G., Roda A. (2014). Berberine and Its Metabolites: Relationship between Physicochemical Properties and Plasma Levels after Administration to Human Subjects. J. Nat. Prod..

[158] Zhang C., Li C., Chen S., Li Z., Jia X., Wang K., Bao J., Liang Y., Wang X., Chen M. (2017). Berberine Protects against 6-OHDA-Induced Neurotoxicity in PC12 Cells and Zebrafish through Hormetic Mechanisms Involving PI3K/AKT/Bcl-2 and Nrf2/HO-1 Pathways. Redox Biol..

[159] Habtemariam S. (2020). Berberine Pharmacology and the Gut Microbiota: A Hidden Therapeutic Link. Pharmacol. Res..

[160] Chen C.-Y., Zhang Y. (2025). Berberine: An Isoquinoline Alkaloid Targeting the Oxidative Stress and Gut-Brain Axis in the Models of Depression. Eur. J. Med. Chem..

[161] Li Y., Ren G., Wang Y.-X., Kong W.-J., Yang P., Wang Y.-M., Li Y.-H., Yi H., Li Z.-R., Song D.-Q. (2011). Bioactivities of Berberine Metabolites after Transformation through CYP450 Isoenzymes. J. Transl. Med..

[162] Feng R., Shou J.-W., Zhao Z.-X., He C.-Y., Ma C., Huang M., Fu J., Tan X.-S., Li X.-Y., Wen B.-Y. (2015). Transforming Berberine into Its Intestine-Absorbable Form by the Gut Microbiota. Sci. Rep..

[163] Simpson C.A., Diaz-Arteche C., Eliby D., Schwartz O.S., Simmons J.G., Cowan C.S.M. (2021). The Gut Microbiota in Anxiety and Depression—A Systematic Review. Clin. Psychol. Rev..

[164] Nikolova V.L., Smith M.R.B., Hall L.J., Cleare A.J., Stone J.M., Young A.H. (2021). Perturbations in Gut Microbiota Composition in Psychiatric Disorders: A Review and Meta-Analysis. JAMA Psychiatry.

[165] Zhu X., Sun Y., Zhang C., Liu H. (2017). Effects of Berberine on a Rat Model of Chronic Stress and Depression via Gastrointestinal Tract Pathology and Gastrointestinal Flora Profile Assays. Mol. Med. Rep..

[166] Huang M., He Y., Tian L., Yu L., Cheng Q., Li Z., Gao L., Gao S., Yu C. (2023). Gut Microbiota-SCFAs-Brain Axis Associated with the Antidepressant Activity of Berberine in CUMS Rats. J. Affect. Disord..

[167] Zhang L., Wu X., Yang R., Chen F., Liao Y., Zhu Z., Wu Z., Sun X., Wang L. (2021). Effects of Berberine on the Gastrointestinal Microbiota. Front. Cell. Infect. Microbiol..

[168] Dalile B., Van Oudenhove L., Vervliet B., Verbeke K. (2019). The Role of Short-Chain Fatty Acids in Microbiota–Gut–Brain Communication. Nat. Rev. Gastroenterol. Hepatol..

[169] Strandwitz P. (2018). Neurotransmitter Modulation by the Gut Microbiota. Brain Res..

[170] Morisaki Y., Miyata N., Nakashima M., Hata T., Takakura S., Yoshihara K., Suematsu T., Nomoto K., Miyazaki K., Tsuji H. (2023). Persistence of Gut Dysbiosis in Individuals with Anorexia Nervosa. PLoS ONE.

[171] Scala M., Tabone M., Paolini M., Salueña A., Iturra R.A., Ferreiro V.R., Alvarez-Mon M.Á., Serretti A., Soltero M.D.R.G., Rodriguez-Jimenez R. (2025). Unlocking the Link Between Gut Microbiota and Psychopathological Insights in Anorexia Nervosa: A Systematic Review. Eur. Eat. Disord. Rev..

[172] Zhao W., Kodancha P., Das S. (2024). Gut Microbiome Changes in Anorexia Nervosa: A Comprehensive Review. Pathophysiology.

[173] Prochazkova P., Roubalova R., Dvorak J., Kreisinger J., Hill M., Tlaskalova-Hogenova H., Tomasova P., Pelantova H., Cermakova M., Kuzma M. (2021). The Intestinal Microbiota and Metabolites in Patients with Anorexia Nervosa. Gut Microbes.

[174] Carbone E.A., D’Amato P., Vicchio G., De Fazio P., Segura-Garcia C. (2020). A Systematic Review on the Role of Microbiota in the Pathogenesis and Treatment of Eating Disorders. Eur. Psychiatry.

[175] Wang Y., Tong Q., Ma S.-R., Zhao Z.-X., Pan L.-B., Cong L., Han P., Peng R., Yu H., Lin Y. (2021). Oral Berberine Improves Brain Dopa/Dopamine Levels to Ameliorate Parkinson’s Disease by Regulating Gut Microbiota. Signal Transduct. Target. Ther..

[176] Tarrago T., Kichik N., Seguí J., Giralt E. (2007). The Natural Product Berberine is a Human Prolyl Oligopeptidase Inhibitor. ChemMedChem.

